# Nipple aspiration and ductal lavage in women with a germline *BRCA*1 or *BRCA*2 mutation

**DOI:** 10.1186/bcr1348

**Published:** 2005-11-14

**Authors:** Gillian Mitchell, Yoland C Antill, William Murray, Judy Kirk, Elizabeth Salisbury, Geoffrey J Lindeman, Juliana Di Iulio, Alvin D Milner, Lisa Devereaux, Kelly-Anne Phillips

**Affiliations:** 1 Division of Haematology and Medical Oncology, Peter MacCallum Cancer Centre, Melbourne, Australia; 2 Research Division, Peter MacCallum Cancer Centre, Melbourne, Australia; 3 Familial Cancer Service, Westmead Hospital, Sydney, Australia; 4 Department of Pathology, Westmead Hospital, Sydney, Australia; 5 Family Cancer Centre, Royal Melbourne Hospital, Melbourne, Australia; 6 Centre for Biostatistics and Clinical Trials, Peter MacCallum Cancer Centre, Melbourne, Australia; 7 Tissue Bank, Peter MacCallum Cancer Centre, Melbourne, Australia

## Abstract

**Introduction:**

The aim of this study was to collect serial samples of nipple aspirate (NA) and ductal lavage (DL) fluid from women with germline *BRCA*1/2 mutations in order to create a biorepository for use in identifying biomarkers of breast cancer risk.

**Methods:**

Between March 2003 and February 2005, 52 women with germline *BRCA*1 or *BRCA*2 mutations (median age 43 years, range 27 to 65 years) were scheduled for six-monthly NA, DL and venesection. DL was attempted for all NA fluid-yielding (FY) and any non-FY ducts that could be located at each visit.

**Results:**

Twenty-seven (52%) women were postmenopausal, predominantly (19/27) from risk reducing bilateral salpingo-oophorectomy (BSO). FY ducts were identified in 60% of all women, 76% of premenopausal women versus 44% of postmenopausal (*P *= 0.026). Eighty-five percent of women had successful DL. Success was most likely in women with FY ducts (FY 94% versus non-FY 71% (*P *= 0.049). DL samples were more likely to be cellular if collected from FY ducts (FY 68% versus non-FY 43%; *P *= 0.037). Total cell counts were associated with FY status (FY median cell count 30,996, range 0 to >1,000,000 versus non-FY median cell count 0, range 0 to 173,577; *P *= 0.002). Four women (8%) had ducts with severe atypia with or without additional ducts with mild epithelial atypia; seven others had ducts with mild atypia alone (11/52 (21%) in total). Median total cell count was greater from ducts with atypia (105,870, range 1920 to >1,000,000) than those with no atypia (174, 0 to >1,000,000; *P *≤ 0.001).

**Conclusion:**

It is feasible to collect serial NA and DL samples from women at high genetic risk of breast cancer, and we are creating a unique, prospective collection of ductal samples that have the potential to be used for discovery of biomarkers of breast cancer risk and evaluate the ongoing effects of risk reducing BSO. DL cellular atypia was not predictive of a current breast cancer and longer follow up is needed to determine whether atypia is an additional marker of future breast cancer risk in this population already at high genetic risk of breast cancer.

## Introduction

Germline *BRCA*1 or *BRCA*2 mutations are associated with a markedly increased lifetime risk of developing breast cancer [1,2] in female carriers. Not all mutation carriers develop breast cancer, and the age of onset of breast cancer remains unpredictable. Decisions regarding breast cancer risk management can therefore be problematic. Better strategies for risk assessment, early detection and prevention of breast cancer are clearly a priority for research; in particular, there is a pressing need for surrogate biomarkers that are highly specific and sensitive indicators of breast cancer risk.

There is a well established association between atypical ductal epithelium identified by histological biopsy [3], nipple aspiration (NA) [4] or fine needle aspiration (FNA) [5] and an increased risk of future breast cancer. The relative risk of developing invasive breast carcinoma for women found to have atypical ductal hyperplasia on breast biopsy is 4.3 times that of the general population and, when combined with a positive family history, the relative risk of invasive breast cancer rises to 9.7 times that of the general population [3]. Women with cellular atypia detected by cytological examination of specimens obtained by NA or FNA have an approximately five-fold higher relative risk of developing breast cancer than women without cellular atypia [4,6,5], which may increase a further six-fold when associated with a family history of breast cancer [4]. Whether the finding of ductal epithelial atypia provides additional prognostic information for women already identified as being at high risk of developing breast cancer by virtue of their *BRCA1/2 *genetic status is not known. Although published reports of NA and FNA have included women at high risk of breast cancer, the proportion of these women who carry a *BRCA1/2 *mutation is unknown and no conclusions can be drawn regarding the utility of these findings specifically in *BRCA1/2 *mutation carriers.

Ductal lavage (DL) can also be used to access the ductal epithelium [7] and promises an improved cell yield over NAF without the more invasive properties of FNA, thus providing better opportunities to identify biomarkers of malignancy. DL also lends itself to repeated sampling of specific ducts, which will enable the monitoring of cytological, biological or molecular changes over time, particularly in response to breast cancer risk-reducing strategies such as bilateral salpingo-oophorectomy (BSO) [8] and chemoprevention [9,10]. DL is unlikely to ever sample a sufficient volume of breast to be useful as a breast cancer detection method (screening tool) [11] but it remains uncertain whether it can sample an adequate volume of the intramammary ductal system to be an efficient breast cancer risk-assessment tool [12,13]. It is important, therefore, that prospective studies investigate whether DL is a useful method to identify biomarkers of breast cancer risk.

Investigations into the potential role of DL as a breast cancer risk-assessment tool have commenced. Epithelial atypia identified by DL is assigned the same clinical significance in the Gail model of risk as atypia in histological tissue sections for determining eligibility for the Study of Tamoxifen and Raloxifene (STAR) trial [14]. It has been suggested that DL can be used to identify women at "high-risk" of breast cancer and who may benefit from tamoxifen chemoprevention [10] by the presence of epithelial atypia [15,16]; this may also facilitate decision-making with regards to risk-reducing surgery. However, there is little firm experimental evidence in support of the routine use of DL as a risk assessment tool at the current time and even less is known about its role in women already at very high risk of breast cancer due to a genetic predisposition to the disease. We initiated a prospective cohort study to collect nipple aspirate fluid (NAF), DL fluid and blood samples from women with germline *BRCA1/2 *mutations with the aim of establishing a biorepository of samples to be used to identify biomarkers of breast cancer risk. Our long-term goal is to examine these samples for ductal epithelial atypia, candidate gene methylation frequency and candidate gene expression by RT-PCR and DNA micro-arrays in addition to ascertaining their proteomic profiles. This report describes our initial experience, with the primary objective being to investigate the practicality of establishing this collection and the nature of the DL samples collected.

## Materials and methods

Between March 2003 and February 2005, eligible women were recruited from three familial cancer centres (Peter MacCallum Cancer Centre, The Royal Melbourne Hospital, Melbourne, and Westmead Hospital, Sydney, Australia), which they had attended for the purpose of genetic testing. Women aged between 25 and 65 years with at least one breast unaffected by cancer and carrying a deleterious germline *BRCA1 *or *BRCA2 *mutation were eligible for the study. Eligible women were invited to participate by letter following permission from their breast specialist. Reasons for exclusion included pregnancy, lactation within the last 12 months, previous subareolar or other surgery that could disrupt the ductal systems, breast implant or breast cancer on the side of the proposed lavage. The study was approved by each Hospital Research Ethics Committee and all participating subjects provided written, informed consent.

Women were scheduled to attend at six-monthly intervals for NA and DL from each breast eligible for the study as well as venesection (for the collection of serum and genomic DNA) for a maximum of three years. Follow up for 10 years following completion of the collection of DL samples with regards breast cancer development is planned. For premenopausal women, we would have preferred to collect samples at the same point in each menstrual cycle but as this proved to be impracticable, details of last menstrual period were obtained for each collection visit. All women were advised to continue with the breast cancer surveillance programme prescribed for them by their breast specialist prior to their participation in this study. At a minimum, this included a recommendation to perform monthly breast self-examination, six-monthly clinical breast examination by their specialist and annual mammography.

### Nipple aspirate fluid collection

After a clinical breast examination, NAF was collected from each breast using the technique as previously described [17]. All NAF collected from a single breast was pooled into 0.5 ml phosphate buffered saline and immediately placed on ice. A 0.25 ml aliquot was immediately frozen at -80°C and the remaining 0.25 ml centrifuged to obtain a cell pellet and supernatant, which were then also stored at -80°C for future studies.

### Ductal lavage collection

DL was performed with the FirstCyte™ DL catheter (Cytyc Corporation, Boxborough, MA, USA) essentially as described [7] with minor modifications. The original DL technique required that only NAF producing ducts were cannulated. As our success rate at obtaining NAF was lower than anticipated, however, we elected to attempt DL in all eligible breasts regardless of NA success. If NAF was produced, attempts were made to cannulate the NAF-producing duct, but if no NAF was produced, DL was performed on ducts identified by gentle probing of the nipple surface with a microdilator (Cytyc Corporation). We found that the procedure was best performed by two operators, one to insert the catheter and massage the breast in the optimal position and the other to operate the inflow and outflow syringes. We defined a successful lavage as one in which a catheter was seated in a duct and permitted free flow of saline into the duct. Cannulated ducts were temporarily marked with a suture placed in the duct orifice and photographed in addition to marking the location of the duct and the radiation pattern of the instilled intraductal anaesthetic on a nipple diagram in order to facilitate repeated cannulation of the same duct on subsequent visits.

All women completed a 'tolerability questionnaire' immediately after the procedure, scoring the intensity of sensation perceived during the lavage procedure on a visual analogue scale of 0 (no discomfort at all) to 10 (the worst pain imaginable) and comparing the intensity of sensation to that experienced during mammography.

### Ductal lavage preparation

Between 10 and 12 ml of DL fluid was recovered from each duct; initially 2 ml was separated into 30 ml Cytolyt™ solution (Cytyc Corporation) for transport to the laboratory for cytological processing and the remainder placed on ice. A significant proportion of our initial samples were considered to have inadequate cellular material for cytological diagnosis (ICMD). Consequently, from September 2003, half the recovered DL fluid was used for cytological analysis. The remaining lavage fluid was temporarily placed on ice and processed within 2 h into cell pellets of approximately 10,000 cells/pellet and supernatants stored separately at -80°C. Total cell counts were obtained using a haemocytometer and Trypan Blue at the start of the handling process.

### Cytological examination

DL samples were processed for cytological examination using the ThinPrep^® ^technique [18], stained by the Papanicolaou method and scored for the proportion of epithelial cells, cytological appearance and epithelial cell atypia by one of us (WM) using the scoring system described in the multi-centre DL study [7]. Briefly, there were five diagnostic categories: inadequate cellular material for diagnosis (ICMD; samples with <10 epithelial cells per slide), benign, mild atypia, severe atypia and malignant. All women were informed of the cytological score. If severe cytological atypia or malignant cells were identified, the woman was referred back to her specialist for repeat clinical breast examination and mammography if this had not been performed in the preceding six months, and breast ultrasound and biopsy of any suspicious lesion. Breast MRI was not performed routinely. Suspicious lesions were managed at the discretion of the treating specialist; when no lesion was identified, the woman was reviewed at three-monthly rather than six-monthly intervals with additional screening investigations performed at the specialist's discretion.

### Data collection and statistical analysis

All data were recorded on study specific case record forms and entered into a database created using Microsoft Access software (Microsoft Corp. Redmond, WA, USA). Data consistency checks were made at the time of data entry and subsequently at the time of statistical analysis. All *BRCA1*/2 mutation carriers registered on the study prior to February 2005 were included in the analysis. Baseline characteristics of these women were summarised in addition to the characteristics of their breasts and cannulated ducts.

Fisher's exact test was used to compare the proportion of NAF-producing breasts, successful cannulations and ICMD ducts, according to both menopausal status and previous history of breast cancer, parity and breast feeding. The nonparametric Mann-Whitney test was used to compare total cell counts (expressed as both cells/ml and total cells) according to menopausal status, NAF-producing status, previous history of breast cancer, parity and breast feeding, development of breast cancer during the study and the presence of DL cytologic atypia. No adjustments have been made for multiple comparisons and all *P* values are two-sided. Statistical analysis was performed using StatXact version 6.0 (Cytel Software Corporation, Cambridge, MA, USA).

## Results

We recruited 52 women with *BRCA1/2 *mutations (Table 1) with a median age of 43 years (range 27 to 65 years). Twenty-seven women (52%) were postmenopausal, many (n = 19) as a consequence of a risk-reducing BSO. Median age at menopause was 45 years (range 30 to 54 years). Twenty-one (40%) had a prior diagnosis of unilateral breast cancer. All but two had received adjuvant chemotherapy and only four received adjuvant therapy with tamoxifen. None of the 31 unaffected women were taking tamoxifen for breast cancer prevention. Consequently, we were unable to investigate separately the effects of different cancer treatments from the previous diagnosis of breast cancer *per se *on NA and DL. Eighty-five percent were parous and seventy-three percent had breast-fed their children.

NA and DL were attempted in all women on at least one occasion; median two visits, range 1 to 5 visits (Table 2). Success of NA was related to menopause status (70% of breasts from premenopausal women had fluid-yielding (FY) ducts versus 38% of breasts from postmenopausal women, *P *= 0.004) but not related to a previous history of contralateral breast cancer (Table 2). Forty-four women (85%) had a successful DL attempt on at least one occasion. Successful ductal cannulation was related to FY status (*P *= 0.008) but not related to menopausal status, previous history of contralateral breast cancer or lactation or parity (Table 2). Current exogenous hormone use had no significant effect on FY status (data not shown), but very few patients were current hormone users.

**Figure 1:**
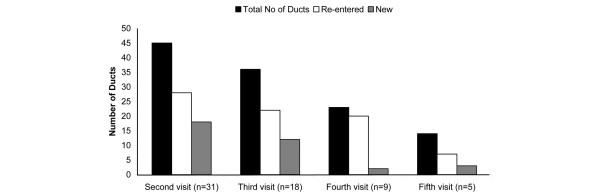
The frequency of individual ducts lavaged on repeat visits per woman. The 'Total number of ducts' equals all ducts ever accessed on at least one previous occasion. 'Repeated ducts' equals ducts re-accessed on that specific visit. 'New ducts' equals a new duct not accessed on a previous visit.

The ability to re-cannulate the same duct on subsequent occasions was determined (Fig. 1). We found that it was more difficult to repeatedly re-cannulate the same duct than we had anticipated, partly due to the close anatomical location of some ducts. For women who attended for more than one lavage appointment, 37%, 39%, 13% and 50% of previously cannulated ducts could not be re-cannulated at each subsequent visit, respectively. By contrast, a proportion of ducts lavaged at each visit were new ducts never previously cannulated, 40%, 33%, 9% and 21% of ducts cannulated on the second, third, fourth and fifth visits, respectively.

### Tolerability

The DL procedure was well tolerated with a median visual analogue scale reading of 2.8/10 (range 0 to 8/10). Three women (6%) had not yet commenced mammographic screening. Of the remainder, 33% of the women thought that the procedure was comparable to the discomfort experienced by mammography, while 33% reported that the procedure caused more discomfort and 29% less discomfort than mammography.

### Cytological analysis

DL samples were more likely to have adequate cellular material for diagnosis if they were collected from FY ducts (*P *= 0.037; Table 3). Allocating 50% of the DL sample for cytological analysis did not reduce the proportion of ICMD samples (data not shown). Similarly, the total cell counts were associated with FY status only (*P *= 0.002). So far, we have found no statistical difference in total counts between the small number (n = 5) of women who developed breast cancer during the study versus women who did not.

**Figure 2:**
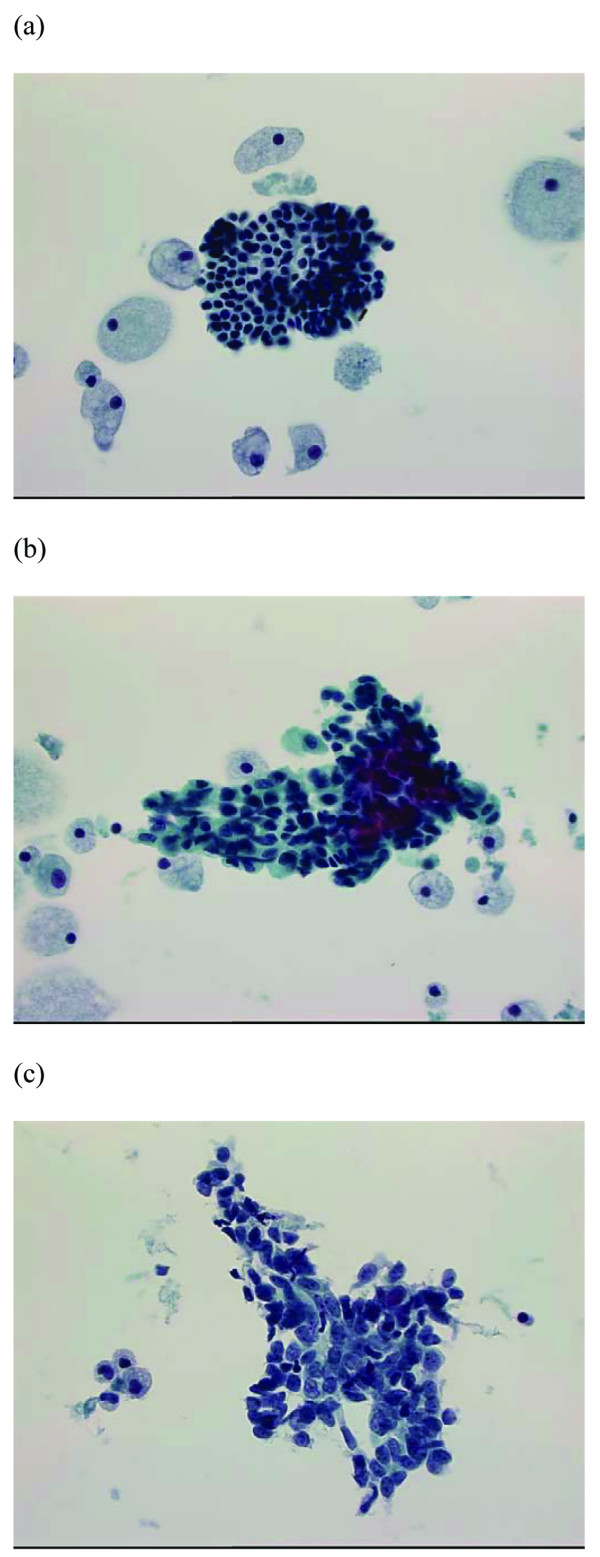
Examples of ductal epithelial cells collected by ductal lavage. **(a) **Benign ductal epithelial cells. **(b) **Epithelial cells with mild atypia. **(c) **Epithelial cells with severe atypia.

Examples of cytological scoring categories are illustrated in Fig. 2. Four women (8%) had severe atypia diagnosed in at least one DL sample, and three of these had mild atypia noted in at least one of their other DL samples. Seven additional women (13%) also had mild atypia alone in at least one DL sample. Overall, there were 20 ducts with atypia from 11 women; 4 of these were classed as severe atypia and 16 as mild atypia. None of the ducts (0/4) with severe atypia were non-FY and two of the ducts with mild atypia were non-FY (2/16). Two of eleven (18%) women with atypia had a previous diagnosis of breast cancer. DL samples with any atypia were significantly more cellular than samples without atypia (*P *< 0.001; Table 3). All of the women with severe atypia had a full clinical assessment by a breast surgeon, including an additional mammogram and breast ultrasound. Three of them also had a breast MRI. No suspicious lesions were identified, but one of them had two fibroadenomas diagnosed by imaging and confirmed by biopsy in the breast with atypia.

Five women, all with *BRCA1 *mutations, were diagnosed with breast cancer (median five months following study entry); the features of their cancers and associated DL samples are summarised in Table 4. Only one cancer was detected through a scheduled annual screening mammogram, and was clinically impalpable at the time of diagnosis. All four other cancers were interval cancers discovered by self-detection of a new breast lump. All prior screening mammograms and clinical breast examinations were reported as normal. One woman had mild cytological atypia in the DL sample from the cancer-containing breast but no cytological atypia was identified in the DLs from the cancer-containing breast of the other three women successfully lavaged. Thus, there appeared to be no correlation between the presence of cytological atypia and the short term development of cancer.

## Discussion

This is the first published study, to our knowledge, to describe the findings from serial NA and DL in a cohort of women with germline mutations in *BRCA1 *and *BRCA2 *genes. We have demonstrated that it is both acceptable and feasible to collect these samples and we have established a prospective collection of samples with ongoing epidemiological data that will be available for future breast cancer biomarker discovery.

We found that we could obtain ductal fluid by NA in 60% of our cohort overall, but that NAF was more often obtained from premenopausal women. Our overall figures are less successful than reported by most (48% to 99%) [19,7,23]. One explanation is that we had a high proportion of postmenopausal women in our cohort (52%) and the proportion of premenopausal women with FY ducts in our cohort is significantly higher than the proportion of postmenopausal women with them. Additional explanations could include the persistence with which the collection of NAF was pursued. For studies of NAF only, it is appropriate to be particularly persistent but in our study, as this was the preliminary to subsequent lavage attempts, we had concerns regarding engorgement of the nipple as a consequence of vigorous attempts to elicit NAF and time constraints were also important. It is possible that if we had been more persistent our overall NAF success rates may have improved a little, although we consider it unlikely that this would have changed our results substantially.

Half of the women in our cohort were postmenopausal despite the median age of only 43 years, predominantly because of risk-reducing BSO (19/27 postmenopausal women). Seventy-six percent of the premenopausal women had FY ducts in contrast to forty-four percent of postmenopausal women. Similar low rates of FY ducts in ovariectomised women have been observed previously; BL King, Yale, found FY ducts in only 23% of women with oophorectomy with or without selective estrogen receptor modulator (SERM) use (personal communication). Conversely, Kurian *et al*. [23] found no effect of menopausal status on the ability to elicit NAF; however, they had a lower overall NAF success rate (48% compared to 60% in our study), which may account for this apparent discrepancy in results. During the course of our study, more women are planning to have a risk-reducing BSO and we will be able to investigate the temporal relationship between NAF production/DL cellularity and BSO.

The ability to re-cannulate the same duct on repeated occasions was less successful than we had anticipated (Fig. 1). Frequently, it was simply that we were unable to locate the duct orifice or, to a lesser extent, the duct was perforated during the cannulation attempt. On most occasions, we could be sure that the same and/or different ducts were cannulated as a consequence of the combined information from the photographs, nipple diagrams and anaesthetic radiation pattern. Despite this information, however, it is possible that we may have re-cannulated the same duct but scored it at a different, although close, location or conversely, that we have recorded a successful re-cannulation but in fact cannulated a different duct due to the close proximity of two duct orifices.

If the DL sample had adequate material for cytological diagnosis (non-ICMD samples), the cellularity was comparable to previous reports [7]. In contrast to previous reports, however, we found that a higher proportion of our samples were categorised as ICMD (39% in contrast to 22% [7]). The higher proportion of ducts with ICMD may be due to a variety of factors. We consider menopausal status to be the most important factor associated with cellularity, but the practice of allocating only a small proportion (initially 20% then later 50%) of the sample, in contrast to previous reports in which the entire lavage sample was used for cytological analysis, may also have contributed to the higher ICMD rate. We found that DL cellularity was related to both FY and menopausal status, which is consistent with the close relationship between these two factors. We are not able to comment on the effect of prior exposure to chemotherapy or tamoxifen or current exogenous hormone use as separate categories on cannulation success rates or sample cellularity as almost all women previously affected by breast cancer had received chemotherapy and few received tamoxifen.

Four women (8%) were identified with severe cytological atypia (in at least one duct on at least one occasion) and 7/52 (13%) with mild cytological atypia, which is consistent with the results from the multicentre DL study (6% and 17% of women had severe or mild atypia, respectively [7]) and from DL studies of other high-risk women in which 23% (7/30) [24] and 28% (17/75) [23] had any atypia (severe or mild). We might have expected to see a higher rate of atypia in our study if the hypothesis that atypia is related to future breast cancer risk is correct, as the women in our study should be at very high risk due to their genetic status. However, the large numbers of women who have undergone risk-reducing BSO in our study may have reduced the overall breast cancer risk in our cohort. The majority of samples with atypia in our study (90%) were from FY ducts, in contrast to 29% reported by Kurian *et al*. [23]. It is difficult to postulate a biological explanation for this discrepancy but it is possible that it is due to a combination of the small numbers of samples with atypia in each study, in addition to the known difficulties in cytological interpretation. All the atypical samples in the Kurian *et al*. study were scored as mildly, rather than severely, atypical, as were most of ours, and it is the differentiation between benign and mildly atypical that is the most debated in cytological scoring.

Five women were diagnosed with breast cancer during our study; one had only mild cytological atypia while the others had no atypia in their DL samples. These women are likely to have had an established malignancy at study entry, although this was not detected by pre-study assessment. Our results are consistent with previous reports that have suggested that both NAF and DL fluid have low sensitivity for the identification of an established malignancy. For NAF, only a low percentage of specimens contain severely atypical or malignant cells, even when the aspirate is obtained from a known cancer-bearing breast (sensitivity 4% [22] to 21% [22,19]). Two studies have reported on the low sensitivity (41% [25], 11% (Wiley, K.: Histology of intraductal lesions – correlation and sampling issues. *Lynn Sage Breast Cancer Symposium, Chicago *2003,) [26]) of DL for established breast cancer. The reasons for such low sensitivity are uncertain, but are possibly related to sampling error by the cannulation of only a limited number of ducts per breast and the blockage/disruption of the ductal system by non-invasive or invasive tumours.

We believe that it is important to be aware of the poor correlation between DL atypia and current breast cancer and that the significance of detecting DL atypia in a high-risk woman is unknown. The finding of no atypia should not reassure a high-risk woman that no malignancy is present and she should be encouraged to continue with regular breast self examination, clinical breast examination and mammography. The finding of atypia requires investigation to exclude malignancy, but we do not consider that there is sufficient evidence currently to consider DL atypia as an additional risk factor in support of prophylactic mastectomy in an otherwise high-risk population.

## Conclusion

Our results demonstrate that DL is an acceptable, minimally invasive method of repeatedly accessing the breast ductal environment, although it requires further validation before it can be established as a breast cancer risk assessment tool, particularly in the high-risk population. It does not appear to be a sensitive breast cancer screening method. We are creating a prospective repository of biologic samples that have the potential to be used to identify biomarkers of breast cancer risk in a high-risk population. Two further cohorts of 50 *BRCA1/2 *mutation carriers each in the UK and US are being recruited using a similar protocol, which will permit a combined analysis of results in the future. In the first two years of our study, five women have developed breast cancer, consistent with published rates of breast cancers in this high-risk group [1,2,27]. If similar rates of breast cancer development are observed in the UK and US centres, it is probable that these samples can be used to identify cytological, biological and molecular biomarkers for breast cancer risk in women with germline *BRCA1/2 *mutations.

## Abbreviations

BSO = bilateral salpingo-oophorectomy; DL = ductal lavage; FNA = fine needle aspiration; FY = fluid yielding; ICMD = inadequate cellular material for diagnosis; NA = nipple aspiration; NAF = nipple aspirate fluid.

## Competing interests

The authors declare that they have no competing interests.

## Figures and Tables

**Table 1:** Characteristics of all study participants

Characteristic	N	%
Total	52	
Age (years; median, range)	43 (27–65)	
*BRCA *mutation		
A1	25	48
A2	27	52
Menopause status		
Premenopausal	25	48
Postmenopausal	27	52
BSO <50 years	19	37
Current use (study entry)		
OCP	3	6
HRT	3	6
Tamoxifen	4	8
Previous diagnosis of breast cancer	21	40
Parous	44	85
Breast fed	38	73

BSO, bilateral salpingo-oophorectomy; HRT, hormone replacement therapy; OCP, oral contraceptive pill.

**Table 2:** Summary of nipple aspiration and ductal lavage episodes

	Women			Eligible breasts		
	N	%	*P*	N	%	*P*

Eligible for study	52			83		
Premenopausal	25	48		43	52	
Postmenopausal	27	52		40	48	
Previous BC	21	40		21	25	
No previous BC	31	60		62	75	
Parous	44	85		68	82	
Non-parous	8	15		15	18	
Breast fed	38	74		60	72	
No breast feeding	14	27		23	28	
FY	31	60		45	54	
Premenopausal	19	76		30	70	
Postmenopausal	12	44	0.026	15	38	0.004
Previous BC	11	52		11	52	
No previous BC	20	65	0.405	34	55	1.000
Parous	25	57		34	50	
Non-parous	6	75	0.450	11	73	0.152
Breast fed	21	55		28	47	
No breast feeding	10	71	0.353	17	74	0.030
No. DL visits (median (range))	2 (1–5)					
Successful DL cannulation	44	85		64	77	
FY	29	94		40	89	
Non-FY	15	71	0.049	24	63	0.008
Premenopausal	22	88		33	77	
Postmenopausal	22	81	0.705	31	78	1.000
Previous BC	17	81		17	81	
No previous BC	27	87	0.700	47	76	0.769
Parous	36	82		53	78	
Non-parous	8	100	0.330	11	73	0.738
Breast fed	32	84		47	78	
No breast feeding	12	86	1.000	17	74	0.772

*P *is for Fisher's exact test. BC, breast cancer; FY, breasts producing nipple aspirate fluid; DL, ductal lavage.

**Table 3:** Summary of associations between patient characteristics and ductal lavage cellularity

	ICMD ducts		Cell count/ml			Total cell count/specimen		
	%	*P*^1^	Median	Range	*P*^2^	Median	Range	*P*^2^

Overall	39		600	0 to 909,090		9481	0 to 21,818,160	
FY Status								
FY duct	32		1625	0 to 909,090		30,996	0 to 21,818,160	
Non-FY duct	57	0.037	0	0 to 4959	<0.001	0	0 to 173,577	0.002
Menopausal status								
Premenopausal	28		1000	0 to 50,000		22,000	0 to 600,000	
Postmenopausal	51	0.034	0	0 to 909,090	0.044	0	0 to 21,818,160	0.055
Previous diagnosis of BC								
Previous BC	31		1000	0 to 909,090		22,000	0 to 21,818,160	
No BC	43	0.361	390	0 to 50,000	0.191	6000	0 to 1,229,667	0.189
Parity								
Parous	35		750	0 to 909,090		12,000	0 to 21,818,160	
Non-parous	58	0.073	0	0 – 50,000	0.395	0	0 to 600,000	0.261
Breast feeding								
Breast fed	31		1000	0 to 909,090		15,426	0 to 21,818,160	
No breast feeding	57	0.024	0	0 to 50,000	0.091	0	0 to 600,000	0.057
BC during study								
BC	20		6666	0 to 25,850		79,992	0 to 542,850	
No BC	40	0.645	500	0 to 909,090	0.133	7688	0 to 21,818,160	0.261
Cytologic atypia								
Mild atypia			5355	84 to 50,000		105,870	1920 to 1,229,667	
Severe atypia			3591	1917 to 18,666	0.75	113,752	48,000 to 447,996	0.892
Atypia (mild + severe)	0		4875	84 to 50,000		105,870	1920 to 1,229,667	
No atypia	50	<0.001	14	0 to 909,090	<0.001	174	0 to 21,818,160	<0.001

Cellularity is expressed both as cells/ml, to account for variations in the volume of DL collected, as well as total number of cells/specimen in order to permit comparisons between our results and other published studies. *P*^*1 *^= Fisher's exact test; *P*^*2 *^= Mann-Whitney test. BC, breast cancer; ICMD, inadequate cellular material for diagnosis; FY, ducts producing nipple aspirate fluid.

**Table 4:** Demographic, tumour and ductal lavage characteristics of women who developed breast cancer after study entry

	Subject				
	PMC13	PMC11	PMC14	PMC15	RMH08

Age (years)	53	51	41	48	37
*BRCA *mutation	A1	A1	A1	A1	A1
Menopause status	Post	Post	Pre	Post	Pre
Prior contralateral breast cancer (age in years)	No	Yes (42)	Yes (35)	Yes (38)	Yes (34)
Previous mammogram (months)	12 (normal)	7 (normal)	5 (normal)	5 (normal)	7 (normal)
Previous CBE (months)	6	1	5	5	7
'Regular' BSE^a^	Yes	Yes	Yes	Yes	Yes
Mode of detection	Mammography	Self	Self	Self	Self
Distance from nipple (cm)	5	3	Not recorded	Not recorded	2
Histological characteristics	Atypical medullary	Lobular	Ductal	Ductal	Ductal
Size (mm)	5.2	20	10, 14, 10 (X3 separate foci)	1.8	5
Grade	3	3	3	3	3
ER status	Negative	Negative	Negative	Negative	Negative
PR status	Negative	Negative	Negative	Negative	Negative
Nodes	Negative	Negative	Negative	Negative	Negative
Ductal lavage					
Previous (months)	5	1	5	12	4
No. ducts cannulated/breast	1 (X1occ)	2 (X1occ)	1 (X2occ)	1 (X1occ)	Unsuccessful
Cell count (duct)/ml	6,666	2,500, 6,666	47,700 + 4,000	15,000	
Total cell count (duct)	79,992	35,000, 93,324	525,555 + 40,000	180,000	
Cytology score	Mild atypia	Benign	Benign	No epithelial cells	

^a^'Regular' as reported by subject (frequency not recorded). BSE, breast self examination; CBE, clinical breast examination; ER, oestrogen receptor; PR, progesterone receptor.
